# The relationship between demographic factors and syncopal symptom in pediatric vasovagal syncope

**DOI:** 10.1038/s41598-023-49722-w

**Published:** 2023-12-20

**Authors:** Shuo Wang, Yali Peng, Runmei Zou, Yuwen Wang, Hong Cai, Fang Li, Xuemei Luo, Juan Zhang, Zhixiang He, Cheng Wang

**Affiliations:** 1 Department of Pediatrics, Xiangya Hospital, Central South University, Changsha, 410008 Hunan China; 2 Department of Pediatric Cardiovasology, Children’s Medical Center, The Second Xiangya Hospital, Central South University, No.139 Renmin Middle Road, Changsha, 410011 Hunan China; 3 Section of Science and Education, The First People’s Hospital of Changde City, Changde, 415000 Hunan China; 4 Department of Pediatrics, Hunan Children’s Hospital, Changsha, 410007 Hunan China

**Keywords:** Paediatric research, Neurological disorders, Cardiovascular biology

## Abstract

This research proposed to retrospectively analyze 20 years of clinical data and investigate the relationship between demographic factors and syncopal symptom in pediatric vasovagal syncope. A total of 2513 children, 1124 males and 1389 females, age range 3–18 years, who presented to Department of Pediatric Cardiovasology, Children's Medical Center, The Second Xiangya Hospital, Central South University with unexplained syncope or pre-syncope and were diagnosed with vasovagal syncope were retrospectively collected and divided into syncope group (n = 1262) and pre-syncope group (n = 1251). (1) Females had a 36% increased risk of syncope compared to males, a 27% increased risk of syncope for every 1-year increase in age, and a 2% decreased risk of syncope for every 1 cm increase in height. (2) A non-linear relationship between age, height, weight and syncope was observed. When age > 10.67 years, the risk of syncope increases by 45% for each 1-year increase in age; when height < 146 cm, the risk of syncope decreases by 4% for each 1 cm increase in height; when weight < 28.5 kg, the risk of syncope decreases by 10% for each 1 kg increase in weight. Demographic factors are strongly associated with syncopal symptom in pediatric vasovagal syncope and can help to predict the risk.

## Introduction

Neurally-mediated syncope (NMS) has been the commonly observed type of syncope, and vasovagal syncope (VVS) is one of its most occurring hemodynamic types. VVS is a clinical disease in which peripheral vascular and cerebral perfusion are inadequate due to nerve reflex-mediated bradycardia and hypotension, leading to transient loss of consciousness or even syncope^1^. Patients may be triggered by tense situation, prolonged standing and sudden change of position (e.g., from lying or sitting or squatting position to upright position). The clinical symptoms of VVS are divided into syncope and pre-syncope. The syncope may be preceded by a series of pre-syncope symptoms such as dizziness, headache, blackness in front of the eyes, nausea, abdominal pain, pallor and sweating. Adolescents and over 60 years old are the two age ranges that have a higher occurrence of syncope^2^. Hu et al^3^. conducted a questionnaire investigation of 4352 children aged 2–18 years in 6 local primary and secondary schools and 3 kindergartens of Changsha and showed that 17.37% of the children had experienced syncope once at least, the average age of occurrence was 13.9 ± 3.1 years, among which the largest number was in the 16-year-old. Sheldon et al^4^. reported the cumulative incidence of VVS by the age of 60 years, and found that 42% of females and 32% of males had 1 episode of VVS at least, and about 1–3% of young children presented with pale faces during syncope. The head-up tilt test (HUTT) has become the gold standard for clinical diagnosis of VVS by passively changing the subject's position to reproduce a VVS attack^5^. Syncope may cause physical injuries of varying degrees, such as abrasions, lacerations, and fractures, to patients according to the environment in which they occur, and the incidence of associated somatic accidental injuries is about 8.8–30%^6–8^. Exploration of syncope-precipitating factors in VVS is beneficial for clinicians to better manage the health of children with VVS, improve the effectiveness of treatment, prevent syncope, and reduce the number of somatic accidental injuries caused by syncope.

Some demographic factors may be correlated with syncope. Guo et al^9^. found that age and sex were risk factors for the occurrence of syncope in middle-aged and elderly people, with older people and females being more likely to experience syncope. Alboni et al^10^. concluded that females had significantly more syncope precursors types as well as number of syncope than males. In previous research by our team, we also found that children with low body mass index (BMI) were more likely to develop situational syncope or postural tachycardia syndrome^11–13^, researchers from other countries have also reported their similar results to our previous findings^14^.

Descriptive research on the demographic factors of pediatric VVS in large samples has not yet been reported. This research proposed to retrospectively analyze 20 years of clinical data on children with VVS in Department of Pediatric Cardiovasology, Children's Medical Center, The Second Xiangya Hospital, Central South University to describe the characteristics of demographic factors in pediatric VVS, to detect possible associations between them and the occurrence of syncope, and to try to quantify their impact efficacy.

## Methods

### Study population

The final diagnosis of VVS was 2513 patients, 1124 males and 1389 females, aged 3–18 years, averaging 11.76 ± 2.83 years. Patients were divided into the syncope and pre-syncope groups based on whether syncope had occurred previously.

Syncope in the grouping of this research was defined as a syncopal episode after experiencing a pre-syncope or a direct syncopal episode, often accompanied by a brief loss of consciousness, collapse, and possible unintentional physical injury. It may be witnessed by bystanders or self-perceived when awake.

Pre-syncope in the grouping of this research was defined as the clinical manifestations of dizziness, headache, blackness before the eyes, nausea, abdominal pain, pallor, and sweating under some conditions or for an unknown reason, but with consciousness and without collapsing or accidental bodily injury.

Informed consent was signed by the subjects themselves or their legal guardians before HUTT. This research was approved by the Medical Ethics Committee of The Second Xiangya Hospital, Central South University, and conforms to the principles stated in the Declaration of Helsinki.

### Inclusion and exclusion criteria

The population included in this research was children who visited the Pediatric Syncope Specialist Outpatient Clinic of The Second Xiangya Hospital, Central South University or were hospitalized in the Pediatric Cardiovascular Specialist Ward of The Second Xiangya Hospital, Central South University between January 2001 and December 2021 due to unexplained syncope and pre-syncope symptoms.

All of the above children underwent physical examination, detailed medical history, 12-lead electrocardiogram (ECG), 24 h electrocardiogram (Holter ECG), echocardiogram, electroencephalogram (EEG), MRI/CT of the head, and fasting glucose, cardiac enzymes, and liver and renal functions, etc., to exclude syncope or pre-syncope caused by cardiac, renal, cerebral, and other organic diseases, immune diseases, and metabolic diseases, and then complete the HUTT.

### Variables

Sex, age, height, weight, heart rate (HR), systolic blood pressure (SBP) and diastolic blood pressure (DBP) at baseline status in HUTT.

### HUTT

The protocol of HUTT was performed according to the previous research^5^. Subjects lay still for 10 min, and then basic HR, blood pressure (BP), and ECG were recorded. Subjects were tilted at 60° head upward. HR, BP and ECG were recorded continuously until either 45 min duration or development of syncope or intolerable near syncopal symptom. If syncope occurred, subjects were rapidly put in the supine position. If syncope or pre-syncope did not occur, tilted posture was maintained, subjects were sublingually medicated with nitroglycerin (4–6 μg/kg, maximum dosage ≤ 300 μg), and HR, BP and ECG were recorded until for 20 min or syncopal symptoms or pre-syncope occurred.

VVS was defined as the development of syncope or pre-syncope accompanied by any of the following responses^5^: (1) hypotension: systolic BP < 80 mmHg, and/or diastolic BP < 50 mmHg, or over 25% decrease in mean BP. (2) bradycardia: HR < 75 bpm for children aged 3–6 years, < 65 bpm for those aged 6–8 years, and < 60 bpm for those older than 8 years. (3) ECG showed sinus arrest, premature junctional contractions. (4) atrioventricular block and asystole ≥ 3 s.

### Statistical analysis

If the continuous variable was normally distributed, it was expressed as mean ± SD and vice versa as the medium (min, max). Categorical variables were expressed in frequency or as a percentage. Chi-square test (categorical variables), Student’s t-test (normal distribution), or Mann–Whitney U-test (skewed distribution) were utilized to analyze differences between pre-syncope group or syncope group. Multiple Logistic regression to analyze the possible association between many factors and syncopal symptom was used and constructed two models to illustrate the stability of this relationship: Model I adjusted for demographic factors (sex, age, height, weight), Model II adjusted for demographic factors and HR, SBP, DBP. A generalized additive model and smooth curve fitting were conducted. If non-linearity was detected, the turning point using a recursive algorithm was calculated and then constructed a two-piecewise Logistic regression on both sides of the turning point. The best fit model based on the *P*-values for the log likelihood ratio test was determined. All the analyses were performed with the statistical software packages R (version 4.1) (http://www.R-project.org, The R Foundation) and EmpowerStats (http://www.empowerstats.com, X & Y Solutions, Inc, Boston, MA). *P*-values < 0.05 (two-sided) were considered statistically significant.

### Ethics approval and consent to participate

Informed consent was signed by the subjects themselves or their legal guardians before HUTT. This research was approved by the Medical Ethics Committee of The Second Xiangya Hospital, Central South University, and conforms to the principles stated in the Declaration of Helsinki.

## Results

### Comparison of basic characteristics between syncope group and pre-syncope group in pediatric VVS and univariate analysis for syncopal symptom

A total of 2513 children with VVS were included in this research, with a mean age of 11.76 ± 2.83 years and 1124 males (44.73%). We found differences in age, height, weight, SBP, DBP and sex between the syncope and pre-syncope groups, and the differences were statistically significant (all *P* < 0.05) **(**Table 1**).**

**Table 1:** Comparison of basic characteristics between syncope group and pre-syncope group in pediatric VVS and univariate analysis for syncopal symptom [Mean ± SD, n (%)].

	Comparison of differences between groups			Univariate analysis for syncopal symptom		
	Pre-syncope group(n = 1251)	Syncope group(n = 1262)	*P*-value	Statistics	OR (95%CI)	*P*-value
Age (years)	11.03 ± 2.47	12.48 ± 2.98	0.000	11.76 ± 2.83	1.21 (1.18, 1.25)	0.000
Height (cm)	146.79 ± 15.10	152.05 ± 15.21	0.000	149.44 ± 15.38	1.02 (1.02, 1.03)	0.000
Weight (kg)	37.79 ± 11.97	42.14 ± 12.67	0.000	39.98 ± 12.52	1.03 (1.02, 1.04)	0.000
HR (bpm)	83.56 ± 16.69	82.42 ± 17.58	0.095	82.99 ± 17.15	1.00 (0.99, 1.00)	0.954
SBP (mmHg)	107.34 ± 10.29	108.49 ± 10.70	0.006	107.92 ± 10.51	1.01 (1.00, 1.02)	0.006
DBP (mmHg)	67.11 ± 7.68	67.79 ± 8.22	0.032	67.45 ± 7.96	1.01 (1.00, 1.02)	0.032
Sex						
Male	620 (49.56)	504 (39.94)	0.000	1124 (44.73)	1.0	
Female	631 (50.44)	758 (60.06)		1389 (55.27)	1.48 (1.26, 1.73)	0.000

The above indicators were further analyzed by a univariate binary regression, we found a positive correlation between age, height, weight, SBP, DBP and syncope, with each 1-unit increase in these indicators leading to a 21%, 2%, 3%, 1% and 1% increase in the risk of syncope, respectively. And we also found a positive correlation between sex and syncope, which means a 48% increase in the risk of syncope in females compared to males (all *P* < 0.05) **(**Table 1**)**.

### Linear relationship between demographic factors and syncopal symptom in different models

To further explore whether the effects between demographic factors and syncopal symptom in univariate analysis existed independently, multiple binary Logistic regressions were constructed to verify their stability. The effects of age and sex on syncope in both models showed excellent stability (< 10% change in OR) compared to the univariate analysis. The effect of height on syncope has changed, showing a trend towards protection. Thus, in the fully adjusted model II for age, the risk of syncope increased by 27% for each 1-year increase in age. In the fully adjusted model II for height, the risk of syncope was reduced by 2% for each 1 cm increase in height (all *P* < 0.01) (Table 2).

**Table 2:** The relationship between age, height, weight and syncopal symptom in different models.

Age				Height				Weight			
Model I		Model II		Model I		Model II		Model I		Model II	
OR (95%CI)	*P*-value	OR (95%CI)	*P*-value	OR (95%CI)	*P*-value	OR (95%CI)	*P*-value	OR (95%CI)	*P*-value	OR (95%CI)	*P*-value
1.27 (1.21, 1.34)	0.000	1.27 (1.21, 1.34)	0.000	0.98 (0.97, 0.99)	0.002	0.98 (0.97, 0.99)	0.002	1.01 (1.00, 1.02)	0.068	1.01 (1.00, 1.03)	0.068

Result variable: syncope or pre-syncope.

Exposure variable: age, height, weight.

Model I in age adjusted for: sex, height, weight.

Model II in age adjusted for: sex, height, weight, HR, SBP, DBP.

Model I in height adjusted for: sex, age, weight.

Model II in height adjusted for: sex, age, weight, HR, SBP, DBP.

Model I in weight adjusted for: sex, age, height.

Model II in weight adjusted for: sex, age, height, HR, SBP, DBP.

In the fully adjusted model II for sex, females had a 36% increased risk of syncope compared to males (*P* < 0.01) (Table 3). Weight failed to maintain the same significance of difference as in the univariate analysis, but a trend towards an independent risk effect was still presumed (OR > 1, *P* = 0.068).

**Table 3:** The relationship between sex and syncopal symptom in different models.

Sex	Model I		Model II	
	OR (95%CI)	*P*-value	OR (95%CI)	*P*-value
Male	1.0	0.000	1.0	0.000
Female	1.36 (1.15, 1.61)		1.36 (1.15, 1.60)	

Result variable: syncope or pre-syncope.

Exposure variable: sex.

Model I in sex adjusted for: age, height, weight.

Model II in sex adjusted for: age, height, weight, HR, SBP, DBP.

### Non-linear relationship between age, height, weight and syncopal symptom

**Figure 1:**
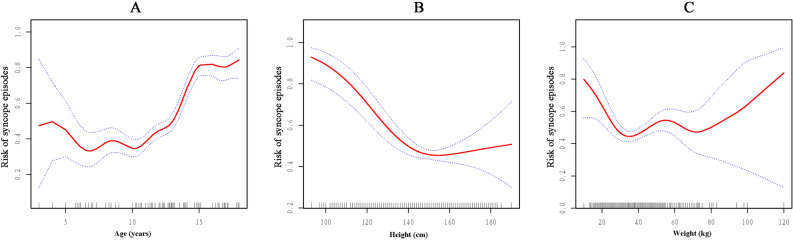
Non-linear relationship between age, height, weight and syncopal symptom. (**A**): Non-linear relationship between age and syncope. (**B**): Non-linear relationship between height and syncope. (**C**): Non-linear relationship between weight and syncope. (**A**) adjusted for: sex, height, weight, HR, SBP, DBP. (**B**): adjusted for: sex, age, weight, HR, SBP, DBP. (**C**): adjusted for: sex, age, height, HR, SBP, DBP.

After determined the independent linear relationship between demographic factors and syncopal symptom, we attempted to further explore whether a nonlinear relationship also existed between them. The results of the smooth curves and the generalized additive model showed that the relationship between age, height, weight and syncopal symptom was nonlinear after adjusting for confounders (Fig. 1).

### Threshold effect analysis of age, height, weight and syncopal symptom using piece-wise Logistic regression

We used both Logistic regression and two-piecewise Logistic regression to fit the association and select the best fit model based on *P*-values for the log likelihood ratio test. Because the *P*-values for the log likelihood ratio test were < 0.05, we chose two-piecewise Logistic regression for fitting the association between age, height, weight and syncopal symptom, because it could accurately represent the relationship.

By two-piecewise Logistic regression and a recursive algorithm, we calculated the turning points for age, height and weight to be 10.67, 146 and 28.5. Which means, when age > 10.67 years, the risk of syncope increases by 45% for each 1-year increase in age; when height < 146 cm, the risk of syncope decreases by 4% for each 1 cm increase in height; when weight < 28.5 kg, the risk of syncope decreases by 10% for each 1 kg increase in weight (all *P* < 0.01) **(**Table 4**)**.

**Table 4:** Non-linear relationship between age, height, weight and syncopal symptom.

	Age		Height		Weight	
	OR (95%CI)	*P*-value	OR (95%CI)	*P*-value	OR (95%CI)	*P*-value
Model I						
One-line slope	1.27 (1.21, 1.34)	0.000	0.98 (0.97, 0.99)	0.002	1.01 (1.00, 1.03)	0.068
Model II						
Turning point (K)	10.67		146		28.5	
< K slope 1	0.94 (0.86, 1.03)	0.205	0.96 (0.94, 0.97)	0.000	0.90 (0.86, 0.95)	0.000
> K slope 2	1.45 (1.36, 1.55)	0.000	1.01 (0.99, 1.03)	0.224	1.01 (1.00, 1.02)	0.099
LRT test	0.000		0.000		0.000	

Result variable: syncope or pre-syncope.

Exposure variable: age, height, weight.

Model II in age adjusted for: sex, height, weight, HR, SBP, DBP.

Model II in height adjusted for: sex, age, weight, HR, SBP, DBP.

Model II in weight adjusted for: sex, age, height, HR, SBP, DBP.

## Discussion

In this research, after retrospectively analyzing the clinical data of children with VVS over a 20-year period, we found an association between demographic factors and syncopal symptom and explored the trend of a more refined syncopal symptom in pediatric VVS under different ranges of demographic factors. In terms of the overall linear relationship, the risk of syncope increased with older age, shorter height, heavier weight, and sex as female. This led us to wonder why the differences in demographic factors mentioned above affect syncopal symptom in pediatric VVS.

We found age was positively associated with syncope in pediatric VVS. By threshold effect we noticed that the effect of age on syncopal symptom in pediatric VVS was mainly concentrated in children above 10.67 years of age. Many physiological parameters of the human body are age-related, such as venous volume and pressure receptor sensitivity, which also vary according to age. Shivaram et al^15^. reported that the pathophysiological mechanisms of syncope change with age. The characteristic high venous volume found in adult syncope occurs at ages older than 12 years, leading to progressive hypotension in the later stages of syncope, whereas children younger than 12 years tend not to have the inferior vena cava dilation associated with syncope. Revasi et al^16^. reported that pressure receptor sensitivity increases progressively with age in children and that the heart becomes more responsive to adrenergic stimulation, making older children more susceptible to syncope. Guo et al^9^. found an increased risk of syncope recurrence in older patients with VVS, and the rate of syncope recurrence also increased with age, especially in female patients. The sensitivity and specificity of predicting syncope recurrence at age 53.5 years or older was 72.7% and 52.7%, respectively.

Height was negatively associated with syncope in pediatric VVS, whereas weight was negatively associated with syncope only within a certain range. In previous research by our team, it was reported that children with NMS with low BMI were prone to syncope symptoms^11–13^. BMI is a composite indicator, affected by the effects from height and weight, the higher the BMI, the higher the degree of blood reflux in the lower extremity extra-fascial veins ^17^, for each 1 kg increase in body weight, SBP increased by 0.77 mmHg (*P* < 0.01)^18^, and with the increased SBP providing perfusion to the brain, the risk of syncope was subsequently reduced. Combined with the results in the threshold effect it is not difficult to observe that this may stem from the dual protective effect of height and weight. The increase in height often brings an increase in hydrostatic pressure, leading to hypertrophy of their cardiac and small arterial walls and their adaptation to the circulatory system, thus preventing sudden changes in the blood supply to the head^19^. The increase in weight strengthens the muscle pump of the lower limbs, providing the necessary energy for venous blood return to the lower limbs, and it also enhances venous contraction and diastole through the squeezing effect, leading to a relatively more cardiopulmonary blood return^20^, which provides blood supply to the brain.

Sex was positively associated with syncope in pediatric VVS, females showed a higher risk than males. This may be related to the existence of sex differences in muscle mass, muscle enzymes, and venous compliance in children. Thijs et al^21^. concluded that males have more muscle mass and stronger muscle pump contraction than females, which provides more blood pooling. Zhang et al^22^. also found that serum creatine kinase (CK) and creatine kinase MB (CK-MB) levels were correlated with sex, with lower CK levels in females than in males in the VVS group. Serum CK-MB levels were also negatively correlated with age, height, weight, and BMI, and positively correlated with HR, and both CK and CK-MB significantly affected VVS occurrence after adjusting for the effects of sex, age, height, weight, BMI, and HR. Skoog et al^23^. reported that reduced venous compliance was an important determinant of postural intolerance in female with VVS. Venous compliance was significantly reduced in female with VVS at low venous pressures and was associated with a maximum lower body negative pressure tolerance index in the low-pressure range. Reduced venous compliance at low venous pressure may adversely affect the mobilization of peripheral venous blood to the central circulation during hypovolemic circulatory stress in females with VVS.

As children grow and increase in height and weight, the risk of syncope should decrease, but age shows an increased risk for syncope. We considered the trend related to the change in height and weight, as age increases, the increase in height and weight gradually slows down at the end of youth and the protective effect on the syncope no longer grows significantly, while the risk effect of age is still increasing, gradually overwhelming the protective effect provided by height and weight, so there are more pieces in older children, and the first peak in the syncope is generally around 16 years of age^3^. We considered that the above may be related to multiple factors such as pressure reflex sensitivity, dilatory response to veins, muscle sympathetic activity, SBP and DBP in different sexes ^24–26^. Beske et al^24^. reported that cardiac vagal pressure reflex gain was significantly lower in females than in males. The response to venous dilatation is diminished in females compared to males, and the increase in cardiac volume per beat is smaller in females than in males^25^. Muscle sympathetic nerve activity (MSNA) increases with age in both females and males, with activity significantly lower in younger females than in males, but accelerating significantly with age^26^.

This research describes the characteristics of demographic factors in pediatric VVS in more detail by analyzing 20 years of clinical data from single center, and through smoothed curve fitting, it allows readers to visualize the trends of risk factors for syncopal symptom in pediatric VVS and quantifies their different independent effects on its. Clinically, focusing on the relationship between demographic factors and syncopal symptom in pediatric VVS provides some data support and theoretical reference for early identification, individualized treatment and health management of syncopal symptom in pediatric VVS.

## Strengths and limitations

This research has a large sample size and a long duration span, thus the results obtained in this research are highly representative and systematically complement the influence of demographic factors on the syncopal symptoms of VVS children, as well as laterally corroborate the results of some previous small-sample studies, which is of strong reference value for clinicians engaged in the research of syncopal disorders in children. However, the samples in this research were all from a single center's data, and no data from other centers were collected as external validation, which may result in the limitation of the research results, and we look forward to the opportunity to combine the data from more centers for validation in the future.

## Conclusions

Demographic factors such as sex, age, height and weight, which are strongly associated with syncopal symptom, are all independent influencers on syncope and can help pediatric syncopal clinicians worldwide to predict the risk of syncope and avoid unintended physical harm.

## Data Availability

The datasets used and/or analyzed during the current study are available from the corresponding author on reasonable request.
